# Supplementary material for: Protocol of the Budapest sleep, experiences, and traits study: An accessible resource for understanding associations between daily experiences, individual differences, and objectively measured sleep
Source: PLoS One. 2023 Oct 19;18(10):e0288909. doi: 10.1371/journal.pone.0288909 (PMC10586695; doi:10.1371/journal.pone.0288909)
Supplement: S1 Text — (DOCX) [file pone.0288909.s001.docx]

**Supplementary Text S1**

A limitation of EEG analyses in BSETS is that EEG data is mainly available from frontal channels. This may limit the validity of analyses of activity with non-frontal topography. For example, if a daily experience affects fast spindles on the subsequent night, this might not be captured by BSETS EEG data because fast spindles occur with a predominantly centro-parietal topography. However, the loss of information may not be total given the considerably similarity between the EEG waveforms of different channels.

In order to quantitatively assess the severity of this limitation, we used scalp EEG data from a large sample of 203 adults [(Ujma et al. 2023)](https://sciwheel.com/work/citation?ids=15007639&pre=&suf=&sa=0&dbf=0) with all-night laboratory polysomnography data with 10 channels. (See the original reference for additional recording details.) We calculated correlations between PSD estimates on two frontal (Fp1, Fp2) and eight other (C3, C4, F3, F4, O1, O2, P3, P4) channels to estimate how well non-frontal PSD estimates are approximated by those available in BSETS. The findings are graphically depicted below.


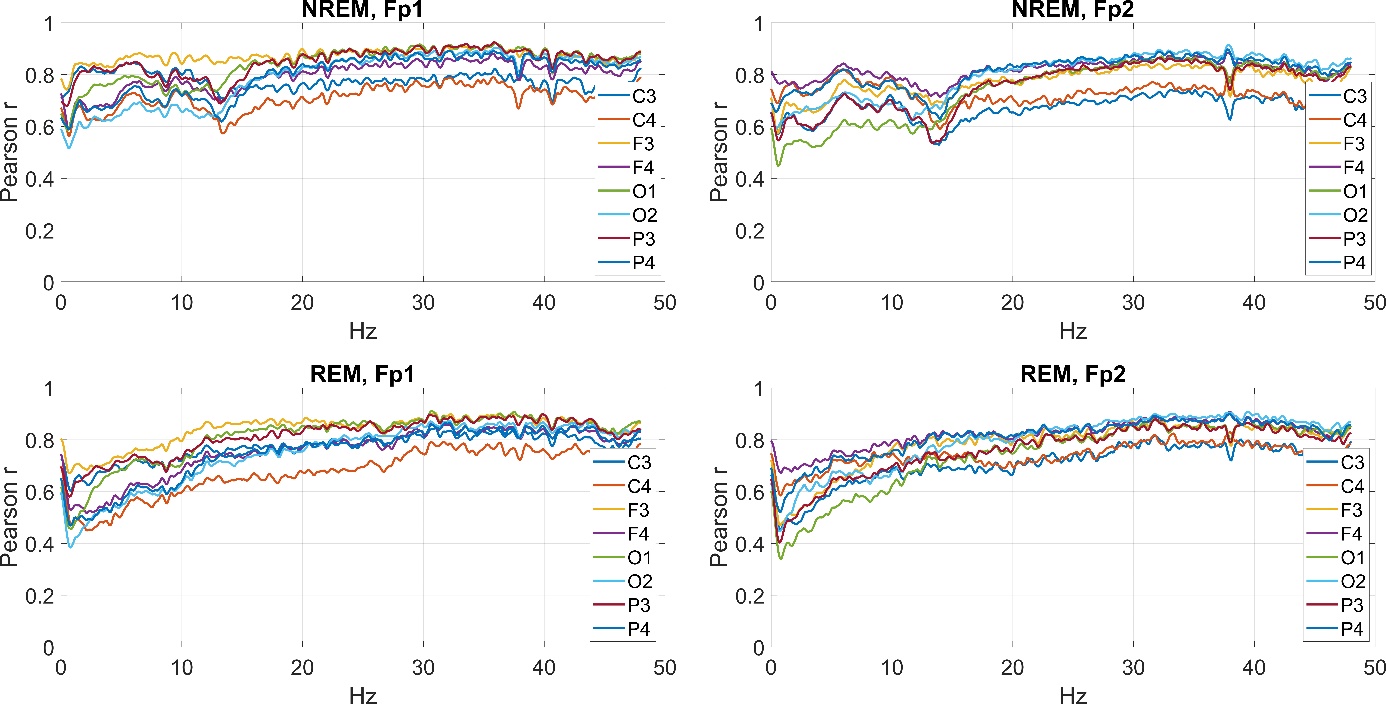


*Between-participant Pearson correlations between PSD estimates obtained from Fp1/F2 and eight other EEG channels.*

We found reasonably high correlations, usually in the order of 0.7-0.8. Local minima are observed at low frequencies in REM (r_min_~0.4), and in the fast spindle frequency range in NREM (r_min_~0.55), in line with the topographic specificity of some EEG waveforms observed at these frequencies (most likely, eye movements and fast spindles).

These findings confirm that frontal PSD estimates are an imperfect, but reasonably accurate proxies of PSD estimates on other channels. Frontal channels in BSETS likely have considerable utility in detecting EEG events with non-frontal maxima.
